# Does donor age older or younger than 70 years influence lung transplantation outcomes?

**DOI:** 10.1016/j.xjon.2025.101555

**Published:** 2025-12-11

**Authors:** Emely Ögren, Jesper Magnusson, Maria Tholén, Emma C. Hansson, Aldina Pivodic, Göran Dellgren

**Affiliations:** a Department of Cardiothoracic Surgery, Sahlgrenska University Hospital, Gothenburg, Sweden; b Department of pulmonary medicine, Institute of medicine, Sahlgrenska University Hospital, Gothenburg, Sweden; c Department of Molecular and Clinical Medicine, Institute of Medicine, Sahlgrenska Academy, University of Gothenburg, Gothenburg, Sweden; d Department of Anesthesiology and Intensive Care, Sahlgrenska University Hospital, Gothenburg, Sweden; e Department Ophtalmology, Sahlgrenska University Hospital, Gothenburg, Sweden; f APNC Sweden, Mölndal, Sweden; g Transplant Institute, Sahlgrenska University Hospital, Gothenburg, Sweden; h Department of Surgery, Institute of Clinical Sciences, Sahlgrenska Academy, University of Gothenburg, Gothenburg, Sweden

**Keywords:** lung transplantation, donor age, extended criteria donor

## Abstract

**Objective:**

For lung transplantation (LTx) we are lacking long-term outcomes using older donors. The aim of our study was to evaluate our cohort regarding the use of donors aged 70 years and older compared with those younger than age 70 years after LTx.

**Methods:**

In a retrospective single-center study including all LTx procedures performed between 2006 and 2024 (n = 689) we compared short- and long-term mortality and retransplantation between groups based on donor age older or younger than 70 years.

**Results:**

Recipients with older donors (n = 75 [10.9%]) were older (60.4 ± 9.5 years vs 52.7 ± 14.1 years; *P* < .0001), shorter (*P* = .024), had a larger proportion with a history of smoking (*P* = .014), and a lower preoperative forced expiratory volume in 1 second (*P* = .011). Older donors were more often women (*P* = .0027), had a shorter donor height (*P* < .0001), and the cause of death was more frequently intracranial hemorrhage (*P* < .0001). One-year mortality was comparable (donors younger than age 70 years: 12.5%, donors aged 70 years and older: 10.7%; *P* = .64). Forced expiratory volume in 1 second at 1 year was higher in recipients with younger donors (*P* = .0052). In a Cox regression analysis recipient age (*P* < .0001), body mass index (*P* = 0.039), donor age per 10 years increase (*P* = .03), and donor age 70 years and older (*P* = .026) were significantly associated with long-term mortality. Overall survival was lower for recipients with older donors (adjusted hazard ratio, 1.44; 95% CI, 1.04-1.97; *P* = .026). After propensity score matching, differences remained with poorer survival in recipients with older donors (hazard ratio, 1.62; 95% CI, 1.02-2.6; *P* = .043).

**Conclusions:**

This study shows that 1-year survival using grafts from donors older than age 70 years is comparable to those of younger donors, but long-term survival is inferior, which indicates that grafts from older donors should be used in selected patients undergoing LTx.

## Graphical Abstract

**Figure:**
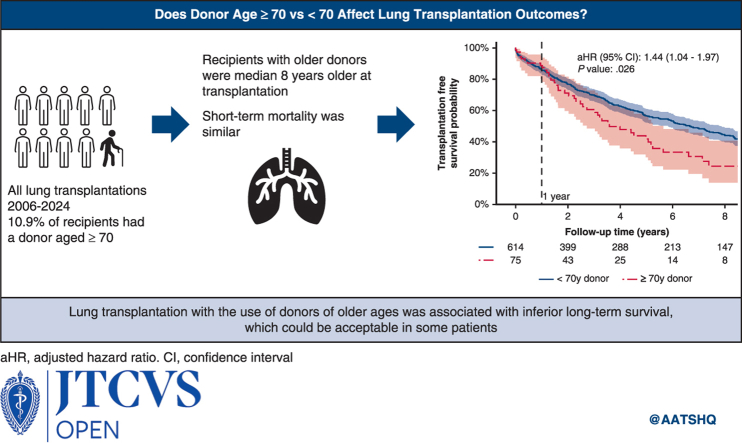

**Figure:**
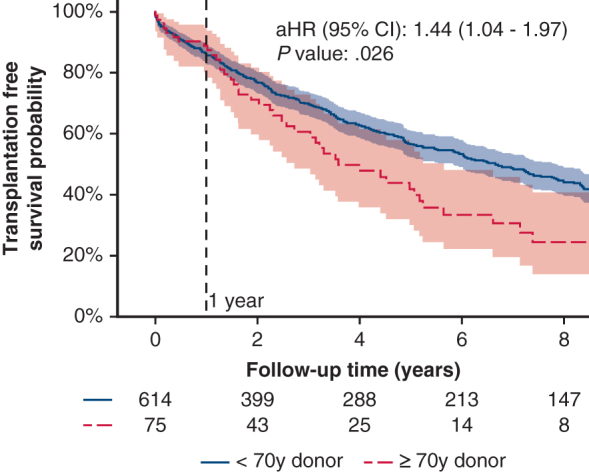
Survival for all-cause mortality, including retransplantation by donor age. Adjusted hazard ratio, 1.44; 95% CI, 1.04-1.97.

**Central Message**

Receiving lungs from donors aged 70 years and older shows inferior long-term retransplantation-free survival and should be considered in patients where these results can be acceptable.

**Perspective**

The use of older donors has been a controversial issue in lung transplantation and previous studies have shown conflicting results. We analyzed outcomes with acceptable short-term survival but inferior long-term survival in comparison to younger donor lungs. Our findings are of significance because they contribute to decision making by clinicians allocating grafts to recipients.

Lung transplantation (LTx) is the ultimate treatment for end-stage respiratory failure. There is an overall shortage of suitable donor grafts, which has led to different strategies to expand the donor pool. The previous criteria for a so-called ideal donor were age younger than 55 years, ABO compatibility, clear chest radiograph, <20 pack-years of smoking, absence of chest trauma or previous cardiopulmonary surgery, Pao_2_ > 300 (inspired oxygen fraction = 1.0, positive end-expiratory pressure = 5 cm H_2_O), no evidence of aspiration/sepsis, absence of purulent secretions at bronchoscopy and absence of organisms on sputum gram stains.^1^

As clinical reality has developed, the use of so-called extended-criteria donors has increased, with age emerging as a factor that is being revised. Previous data are rather conflicting with some studies indicating inferior survival using older donors,^2^, ^3^, ^4^ whereas other studies imply that using donors older than age 70 years has no influence on survival.^5^, ^6^, ^7^, ^8^ A study using the United Network of Organ Sharing database by Hayes and colleagues^9^ reflected worse outcomes in younger patients receiving older donor lungs, thus promoting age-matching when selecting donors.

The purpose of our study was to evaluate our cohort over the past 18 years of LTx comparing outcomes when using donors aged 70 years or older compared with younger than age 70 years. The primary end point was overall retransplantation-free survival.

## Methods

### Study Design and Study Population

At our center, LTx has been performed since 1990 (n = 1031, September 30, 2024) and the first transplantation using a donor aged 70 years or older was performed during June 2006. We conducted a retrospective single-center study, including all LTx procedures performed between June 2006 and September 2024 (n = 737; mean age, 53.4 ± 13.9 years; 48.2% women, donor aged 70 years or older, 10.3%). We excluded combined heart-lung transplantations. Final date of follow-up was September 2024.

For more reliable statistical evaluations, patients were included only for their first LTx during the study period. All re-LTx procedures for the same patients were excluded because the primary end point was death or retransplantation. In our cohort 48 (6.5%) underwent retransplantation during the study period. The recipients included in our study (n = 689) were categorized into 2 groups based on donor age, a younger group (younger than age 70 years, n = 614) and an older group (aged 70 years or older, n = 75). Baseline characteristics were compared as shown in Table 1.

**Table 1:** Preoperative recipient characteristics overall and by donors younger than age 70 years and aged 70 years and older

Characteristic	Overall (N = 689)	<70 y donor (n = 614)	≥70 y donor (n = 75)	*P* value
Age (y)	53.6 ± 13.9 57.65 (7.76-74.31) n = 689	52.7 ± 14.1 56.69 (7.76-74.31) n = 614	60.4 ± 9.5 63.13 (15.77-72.94) n = 75	<.0001
Sex				.11
Male	354 (51.4)	322 (52.4)	32 (42.7)	
Female	335 (48.6)	292 (47.6)	43 (57.3)	
Weight (kg)	68.5 ± 15.8 68 (7-113) n = 685	68.7 ± 16.0 68 (7-113) n = 610	66.9 ± 13.9 67 (39-104) n = 75	.35
Height (cm)	170.0 ± 10.3 170 (119-195) n = 688	170.3 ± 10.4 170 (119-195) n = 613	167.8 ± 8.5 167 (145-184) n = 75	.024
BMI	23.6 ± 4.5 23.34 (4.94-34.65) n = 685	23.5 ± 4.5 23.33 (4.94-34.65) n = 610	23.8 ± 4.9 23.42 (14.88-32.72) n = 75	.62
Blood type				.85
O	249 (36.3)	218 (35.6)	31 (41.9)	
A	334 (48.7)	305 (49.8)	29 (39.2)	
B	76 (11.1)	65 (10.6)	11 (14.9)	
AB	27 (3.9)	24 (3.9)	3 (4.1)	
Missing	3	2	1	
Diagnosis				.24
COPD	154 (22.4)	127 (20.8)	27 (36.0)	
Alpha-1 antitrypsin deficiency	66 (9.6)	61 (10.0)	5 (6.7)	
Other	80 (11.6)	77 (12.6)	3 (4.0)	
Pulmonary vascular disease	43 (6.3)	38 (6.2)	5 (6.7)	
Cystic fibrosis	51 (7.4)	51 (8.3)	0 (0.0)	
Pulmonary fibrosis	240 (34.9)	210 (34.3)	30 (40.0)	
Retransplantation	19 (2.8)	17 (2.8)	2 (2.7)	
Other interstitial lung disease	34 (4.9)	31 (5.1)	3 (4.0)	
Missing	2	2	0	
Smoking				.014
No	301 (43.8)	278 (45.4)	23 (30.7)	
Past use within 6 mo	2 (0.3)	2 (0.3)	0 (0.0)	
Past use >6 mo	384 (55.9)	332 (54.2)	52 (69.3)	
Missing	2	2	0	
Respirator preoperative				.17
No	653 (95.6)	579 (95.2)	74 (98.7)	
Yes	30 (4.4)	29 (4.8)	1 (1.3)	
Missing	6	6	0	
ECMO preoperative				.69
No	654 (95.1)	582 (94.9)	72 (96.0)	
Yes	34 (4.9)	31 (5.1)	3 (4.0)	
Missing	1	1	0	
Dialysis				.52
No	679 (99.3)	605 (99.3)	74 (98.7)	
Yes	5 (0.7)	4 (0.7)	1 (1.3)	
Missing	5	5	0	
Last urgency				.64
Patient on extra-corporeal circulatory support	18 (2.6)	17 (2.8)	1 (1.3)	
Urgent	13 (1.9)	11 (1.8)	2 (2.7)	
Not marked as urgent	658 (95.5)	586 (95.4)	72 (96.0)	
FEV1 (L)	1.2 ± 0.7 1.00 (0.26-4.50) n = 641	1.3 ± 0.7 1.05 (0.26-4.50) n = 571	1.0 ± 0.6 0.89 (0.30-2.50) n = 70	.011
Percent of expected FEV1 (%)	38.3 ± 19.8 33.13 (6.55-110.01) n = 641	38.7 ± 20.0 33.44 (6.55-110.01) n = 571	35.6 ± 18.1 28.58 (11.69-92.93) n = 70	.26
6-min walking test distance	290.4 ± 132.4 276.00 (14.00-700.00) n = 614	294.4 ± 134.3 286.00 (14.00-700.00) n = 548	257.2 ± 110.3 242.50 (40.00-510.00) n = 66	.013
Waiting d, active	107.3 ± 153.8 53 (0-1638) n = 688	107.2 ± 158.6 51 (0-1638) n = 614	107.5 ± 106.9 74 (1-463) n = 74	.11

Values are presented as mean ± SD, median (range), or n (%), *BMI*, Body mass index; *COPD*, chronic obstructive pulmonary disease; *ECMO*, extracorporeal membrane oxygenation; *FEV1*, forced expiratory volume in 1 s.

Intraoperative and postoperative characteristics are shown in Table 2. The primary end point of overall retransplantation-free survival was additionally described at short-term at 30 days, 90 days, and 1 year. Donor characteristics are shown in Table 3.

**Table 2:** Intraoperative and postoperative characteristics overall and by donors younger than age 70 years and aged 70 years and older

Characteristic	Overall> (N = 689)	<70 y donor (n = 614)	≥70 y donor (n = 75)	*P* value
LTx procedure				.45
Single LTx	125 (18.1)	109 (17.8)	16 (21.3)	
Double LTx	564 (81.9)	505 (82.2)	59 (78.7)	
Ischemic time for first lung (min)	252.9 ± 104.2 241 (75-978) n = 579	253.8 ± 107.7 241 (75-978) n = 511	246.3 ± 73.3 237 (98-439) n = 68	.93
MCS during LTx				.53
No	465 (67.5)	412 (67.1)	53 (70.7)	
Yes	224 (32.5)	202 (32.9)	22 (29.3)	
Graft reduction				.65
No	674 (98.0)	600 (97.9)	74 (98.7)	
Yes	14 (2.0)	13 (2.1)	1 (1.3)	
Missing	1	1	0	
Ventilator time (h)	4.9 ± 13.5 0 (0-127) n = 684	5.2 ± 13.9 0 (0-127) n = 610	2.9 ± 8.9 0 (0-54) n = 74	.015
ECMO after LTx				.14
No	671 (97.5)	596 (97.2)	75 (100.0)	
Yes	17 (2.5)	17 (2.8)	0 (0.0)	
Missing	1	1	0	
Total d in ICU postoperative	8.3 ± 13.1 3 (0-106) n = 689	8.4 ± 13.5 3 (0-106) n = 614	6.8 ± 9.1 3 (1-48) n = 75	.88
FEV1 after 1 y (L)	2.3 ± 0.9 2.2 (0.6-5.0) n = 504	2.3 ± 0.9 2.2 (0.6-5.0) n = 450	1.9 ± 0.7 1.9 (0.8-3.3) n = 54	.0052
Percent of expected FEV1 after 1 y (%)	71.9 ± 25.3 70.6 (17.7-149.1) n = 504	72.3 ± 25.4 71.4 (17.7-149.1) n = 450	68.4 ± 23.8 69.1 (29.5-123.8) n = 54	.30
PGD grade 3 at 72 h				.32
No	661 (98.8)	587 (98.7)	74 (100.0)	
Yes	8 (1.2)	8 (1.3)	0 (0.0)	
Missing	20	19	1	
30-d mortality				.55
No	662 (96.1)	589 (95.9)	73 (97.3)	
Yes	27 (3.9)	25 (4.1)	2 (2.7)	
90-d mortality				.78
No	648 (94.0)	578 (94.1)	70 (93.3)	
Yes	41 (6.0)	36 (5.9)	5 (6.7)	
1-y mortality				.64
No	604 (87.7)	537 (87.5)	67 (89.3)	
Yes	85 (12.3)	77 (12.5)	8 (10.7)	

Values are presented as n (%), mean ± SD, or median (range). *LTx*, Lung transplantation; *MCS*, mechanical circulatory support; *ECMO*, extracorporeal membrane oxygenation; *ICU*, intensive care unit; *FEV1*, forced expiratory volume in 1 s; *PGD*, primary graft dysfunction.

**Table 3:** Donor characteristics overall and by donors younger than age 70 years and aged 70 years and older

Characteristic	Overall (N = 689)	<70 y donor (n = 614)	≥70 y donor (n = 75)	*P* value
Donor age (y)	49.4 ± 16.7 52 (7 to 77) n = 689	46.6 ± 15.6 49 (7 to 69) n = 614	72.3 ± 2.0 72 (70 to 77) n = 75	<.0001
Donor sex				.0027
Male	333 (48.3)	309 (50.3)	24 (32.0)	
Female	356 (51.7)	305 (49.7)	51 (68.0)	
Donor body weight (kg)	74.8 ± 16.3 74 (23 to 160) n = 686	75.0 ± 16.4 74 (23 to 160) n = 611	73.3 ± 15.3 70 (48 to 140) n = 75	.42
Donor height (cm)	172.1 ± 10.0 172 (130 to 195) n = 686	172.5 ± 10.1 172 (130 to 195) n = 611	168.1 ± 8.3 167 (150 to 192) n = 75	<.0001
BMI	25.2 ± 4.8 25 (14 to 63) n = 685	25.1 ± 4.8 25 (14 to 63) n = 610	25.8 ± 4.0 25 (18 to 41) n = 75	.23
Blood type				.32
O	304 (45.4)	265 (44.6)	39 (52.0)	
A	286 (42.8)	259 (43.6)	27 (36.0)	
B	70 (10.5)	61 (10.3)	9 (12.0)	
AB	9 (1.3)	9 (1.5)	0 (0.0)	
Missing	20	20	0	
Donor cause of death				<.0001
Spontaneous intracranial hemorrhage	389 (56.5)	330 (53.7)	59 (78.7)	
Cerebral infraction	32 (4.6)	26 (4.2)	6 (8.0)	
Brain tumor	1 (0.1)	1 (0.2)	0 (0.0)	
Traumatic brain injury	63 (9.1)	57 (9.3)	6 (8.0)	
Cerebral anoxia after cardiorespiratory arrest	76 (11.0)	76 (12.4)	0 (0.0)	
CNS infection	7 (1.0)	7 (1.1)	0 (0.0)	
Other causes of death	121 (17.6)	117 (19.1)	4 (5.3)	
Donor smoking				.58
No	216 (59.3)	181 (57.3)	35 (72.9)	
Yes	94 (25.8)	92 (29.1)	2 (4.2)	
Past use	54 (14.8)	43 (13.6)	11 (22.9)	
Missing	325	298	27	
Donation after circulatory death				.49
No	670 (97.2)	598 (97.4)	72 (96.0)	
Yes	19 (2.8)	16 (2.6)	3 (4.0)	
Donor time in ICU (d)	3.1 ± 3.0 2 (−1 to 33) n = 678	3.2 ± 3.1 2 (−1 to 33) n = 607	2.7 ± 1.8 2 (1 to11) n = 71	.60

Values are presented as mean ± SD, median (range), or n (%). *BMI*, Body mass index; *CNS*, central nervous system; *ICU*, intensive care unit.

### Statistical Analyses

Continuous variables are presented as mean ± SD and median (minimum and maximum), and categorical variables as frequency with percentage. For tests between donor age groups Fisher exact test was used for dichotomous variables, Mantel-Haenszel χ^2^ trend test for ordered categorical variables, χ^2^ test for nonordered categorical variables, and Mann-Whitney *U* test for continuous variables. Predictors for mortality were determined using Cox regression.

We first investigated the significance of recipients' and donors' background data using a Cox proportional hazards model, adjusted for sex and age (Table E1). Variables that showed an association with a significance level of at least *P* = .10 were considered potential predictors and were subsequently included in a multivariable model. Backward selection was then applied, using a significance threshold of *P* = .05, to refine the final model (Table 4). Additionally, a sensitivity analysis was performed on 1:1 propensity score-matched groups, using recipients' and donors’ background variables. As a matching algorithm, a 1:1 nearest neighbor matching, with the optimal caliper width of 0.2 of the SD of the logit of the propensity score, was used. All tests were 2-tailed. No correction for multiple testing was performed. All analyses were performed using SAS software version 9.4 (SAS Institute Inc).

**Table 4:** Multivariable Cox regression for time to death (including retransplantation)

Variable	Hazard ratio (95% CI)
Age per 10-y increase	1.17 (1.07-1.28) *P* = .0004
BMI per 1-point increase	1.02 (1.00-1.05) *P* = .037
Donor age ≥70 vs <70 y	1.39 (1.01-1.91) *P* = .044

*BMI*, Body mass index.

## Ethical Approval

The study was approved by the research ethics board at the Sahlgrenska Academy, University of Gothenburg, Sweden, following the Helsinki Declaration (diary No. 728-12, amendment 2020-04281, approved September 1, 2020). Patient consent was waived by the research ethics board.

## Results

### Study Population

**Figure 1:**
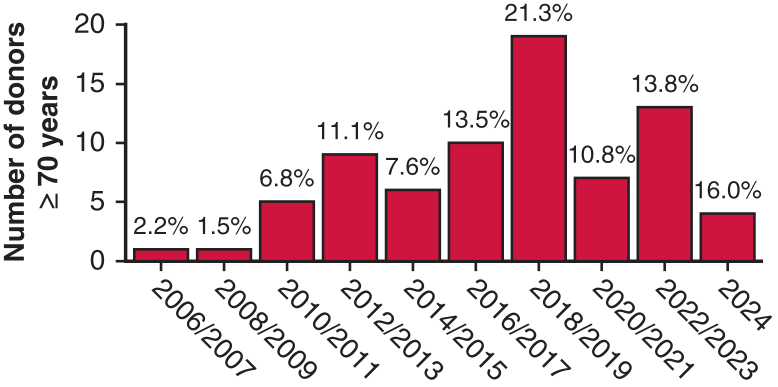
Proportion (%) of lung transplant recipients with donors younger than age 70 years versus age 70 years or older annually from 2006 to 2024. For 2024, inclusion ends in September.

Out of the 689 patients undergoing LTx, 75 (10.9%) had a donor aged 70 years or older. Figure 1 shows the proportion of donors aged 70 years or older used for LTx annually since 2006. Median (minimum-maximum), follow-up time was 3.5 years (0-17.9 years).

The recipients in the group receiving older donor grafts were significantly older (60.4 ± 9.5 vs 52.7 ± 14.1 years; *P* < .0001), had a larger proportion with a history of smoking (69.3% vs 54.6%; *P* = .014), were shorter (167.8 ± 8.5 vs 170.3 ± 10.4 cm; *P* = .024), had a shorter 6-minute walking test distance (257.2 ± 110.3 vs 294.4 ± 134.3 m; *P* = .013) and had a lower forced expiratory volume in 1second (FEV1) (1.0 ± 0.6 vs 1.3 ± 0.7 L; *P* = .011) before transplantation.

### Donors

The mean age of the older donors was 72.3 ± 2.0 years and the younger donors was 46.6 ± 15.6 years. The older donors were more often women (68.0% vs 49.7%; *P* = .0027), were shorter (168.1 ± 8.3 vs 172.5 ± 10.1 cm; *P* < .0001) and the cause of death was more frequently intracranial hemorrhage (78.7% vs 53.7%; *P* < .0001).

### Early and Intermediate Outcomes of Recipients

Postoperative duration of mechanical ventilation was shorter among the recipients with older donors (2.9 ± 8.9 vs 5.2 ± 13.9 hours; *P* = 015); however, the FEV1 at 1 year was lower (1.9 ± 0.7 vs 2.3 ± 0.9 L; *P* = .0052). Crude 1-year mortality and re-transplantation compared between recipients with older or younger donors were similar (10.7% vs 12.5%; *P* = .64).

### Late Outcome of Recipients

**Figure 2:**
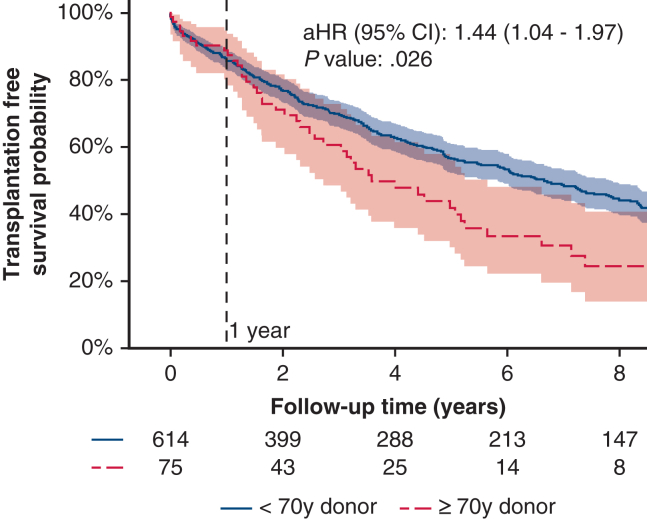
Kaplan-Meier plot for all-cause mortality, including retransplantation by donor age (younger than age 70 years vs age 70 years or older) with indication line at 1 year after lung transplant. Shaded areas denote 95% CI. Adjusted hazard ratio (aHR) was adjusted for age and sex. aHR, 1.44; 95% CI, 1.04-1.97; *P* = .026.

**Figure E1:**
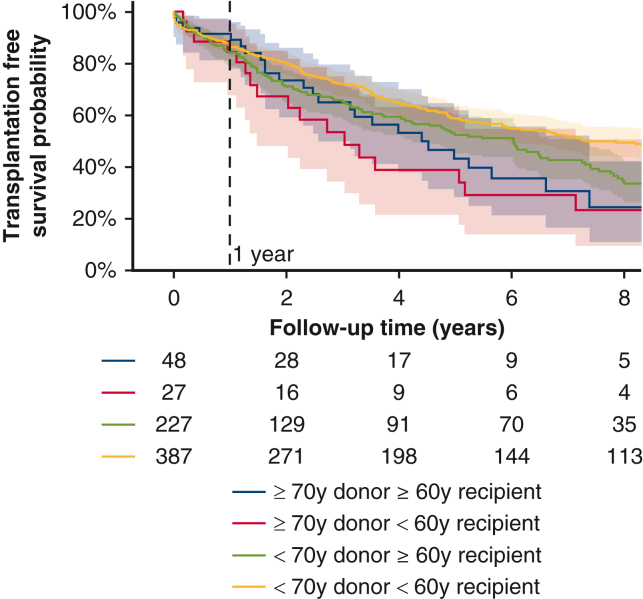
Kaplan-Meier Plot for all-cause death, including retransplantation by group based on donor and recipient age (95% CI).

Long-term outcome (death or retransplantation) is shown in Figure 2. Recipients with older donors had a significantly higher rate of long-term mortality or retransplantation (adjusted hazard ratio [aHR], 1.44; 95% CI, 1.04-1.97; *P* = .026). We then divided the recipients into age groups of younger than age 60 years and age 60 years or older. The long-term outcomes are displayed as a Kaplan-Meier plot in Figure E1. When older donors were further stratified into recipients older or younger than age 60 years, numbers became small and there was no significant difference between groups.

### Cox Regression Analyses

An adjusted Cox regression test was performed to evaluate variables associated with death and retransplantation and is shown in Table E1. Statistically significant predictors were recipient age per 10-year increase (HR, 1.23; 95% CI, 1.13-1.34; *P* < .0001), body mass index per 1-unit increase (HR, 1.0; 2 95% CI, 1.00-1.05; *P* = .039), donor age per 10-year increase (HR, 1.08; 95% CI, 1.01-1.15; *P* = .03) and donor age 70 years or older versus younger than age 70 years (HR, 1.44; 95% CI, 1.04-1.98; *P* = .026). A multivariable Cox regression analysis of time to death or retransplantation was conducted showing that recipient age per 10-year increase (HR, 1.17; 95% CI, 1.07-1.28; *P* = .0004), recipient body mass index per 1-point increase (HR, 1.02; 95% CI, 1.00-1.05; *P* = .037) and donor age 70 years or older versus younger than age 70 years (HR, 1.39; 1.01-1.91; *P* = .044) were significantly and independently associated with outcome (Table 4).

### Propensity Score Matching

**Figure 3:**
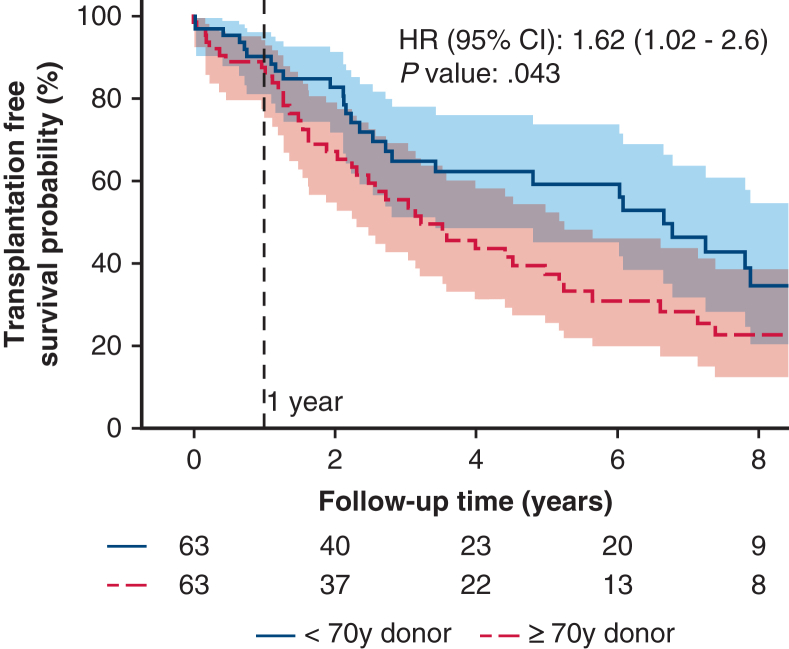
Kaplan-Meier plot for all-cause mortality, including retransplantation by donor age (younger than age 70 years vs age 70 years and older) using 1:1 propensity score-matched groups with indication line for 1 year after lung transplant. Shaded areas denote 95% CI. Adjusted hazard ratio (aHR) was adjusted for age and sex. aHR, 1.62; 95% CI, 1.02-2.6; *P* = .043.

**Figure E2:**
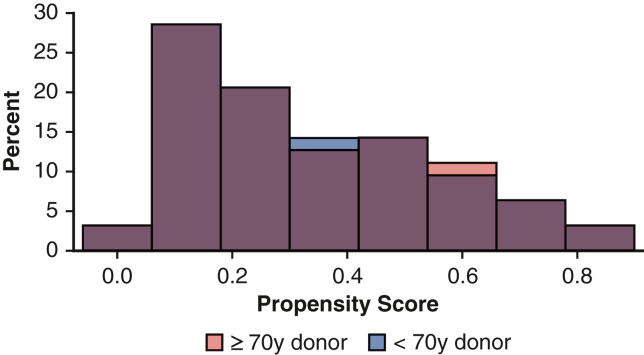
Propensity score after matching (younger than age 70 years vs age 70 years and older).

After propensity score matching, 2 similar groups (n = 63) were created and the descriptive statistics of baseline characteristics for the matched groups are presented in Table E2. Propensity score after matching is displayed in Figure E2. The only factor not sufficiently matched between the groups was recipient pretransplant FEV1 (1.3 ± 0.7 in the younger group vs 1.1 ± 0.6 in the older group; *P* = .07). After propensity score matching, the survival benefit for recipients with younger donors remained significant (HR, 1.62; 95% CI, 1.02-2.6; *P* = .043) (Figure 3).

## Discussion

We have identified that donor age older or younger than 70 years does matter, with significantly worse retransplantation-free survival among LTx recipients receiving older donor grafts. However, there is no survival difference seen during the first year, but it becomes inferior over the subsequent years. This difference should not be interpreted to suggest that grafts from donors older than age 70 years should not be used, but meticulous recipient selection is of importance.

There is an ongoing debate whether grafts from older donors are acceptable or not for Ltx. Among the arguments is that without the use of older grafts many recipients would not be transplanted, due to donor shortage. An ageing lung is known to be susceptible to more injuries, cellular senescence, and lower elasticity,^10^ which has previously discouraged their use in transplantation. In contrast, Lak and colleagues^11^ described steadily improved lung function among 70-year-olds in the general population, which would advocate for using these lungs in the current era in contrast to some decades ago. Our cohort of transplantations over the past 18 years contribute to the real-world evaluation of the use of older donors in LTx. Our results show noninferior survival during the first year after transplantation with lungs from donors older than age 70 years; however, long-term survival is worse among recipients of older grafts. We do not have the information regarding donors when it comes to all different aspects of a so-called ideal donor and there could be confounding in this aspect. A study from Hecker and colleagues^12^ demonstrated noninferior survival and rate of primary graft dysfunction using donors older than age 70 years. They also found that the donor characteristics differed in terms of smoking history and Pao_2_/fraction of inspired oxygen, in favor of the older donor group. This also highlights that careful consideration of older lungs might be of importance in the allocation process.

In the International Society for Heart and Lung Transplantation consensus document for the selection of LTx candidates, there is a suggestion to consider using lungs from older donors in older recipients to facilitate utility and justice of organ transplantation with scarce resources, an idea with which we fully agree.^10^ At our center, a previous study compared donors older and younger than age 55 years in 212 patients and found no difference in survival either among younger or older recipients.^13^ Another study, by Katsnelson and colleagues,^14^ showed no differences in short- or intermediate-term mortality when using older lungs in older recipients and propose using age matching to a wider extent. Based on our findings we do not dissuade from the use of donors of older ages but instead propose nuancing the matching of donor and recipient as well as potentially managing these recipients differently. Also, putting survival in perspective, especially for an age-matched recipient to receive donor lungs older than age 70 years, achieving a 5-year survival of 40% to 50% may very well be a favorable outcome compared with the options for an age-matched recipient.

The age of LTx recipients is increasing in line with the general population life expectancy and currently more than 30% of LTx recipients in the United States are older than age 65 years according to the abovementioned consensus document from the International Society for Heart and Lung Transplantation.^15^ Another publication found no difference in long-term survival of recipients when they were stratified on donor age older and younger than 55 years of age investigated in a larger Scandinavian cohort using Scandia transplant data.^16^ However, in that publication, survival was worse for patients with cystic fibrosis receiving donors older than age 55 years, a signal that donor age makes a difference at least among young recipients. Now, in this single-center analysis, we stratified survival on donor age older or younger than 70 years of age. The mean age was older among recipients receiving grafts from donors older than age 70 years, indicating an appropriate allocation of older grafts to elderly recipients. Although long-term outcome is worse for patients receiving lungs from donors older than age 70 years, we must take into consideration the fact that if we were to limit the graft selection to ideal donors, this would mean that many patients would not be transplanted at all. The median age of our donor cohort was 52 years, which is considerably higher than numbers from North America, which lie around 33 years but in line with the European median age of 51 years for donors.^17^

The 1-year mortality in our cohort was similar to a recent study by Román and colleagues. Their long-term mortality results were superior to ours; however, the number of patients at risk after a few years were very low and thus the results need to be cautiously interpreted.^7^ In our cohort, we found a shorter 6-minute walking test distance in the recipients receiving older grafts, implying that those recipients might have been frailer. To account for differences between groups, we performed a propensity score matching, which did not remove the significant difference in long-term mortality, but there may very well be additional confounders for which we were not able to adjust. Saddoughi and colleagues^18^ recently published a study with 1600 LTxs, of which 98 had donors older than age 70 years. In their Cox regression model donor age was significant in the univariate model but not in the multivariate, thus indicating that there are several factors influencing outcomes when performing a retrospective analysis. In the same study they also found no difference in outcome related to donor age when comparing groups of recipients being either aged 30 to 59 years or older than age 60 years, implying that the younger recipient group had more frail and urgent patients; therefore, the survival was possibly due to recipient factors, not the mismatch in age between recipient and donor.^18^ Hall and colleagues^19^ found, using the United Network of Organ Sharing database, worse survival using donor grafts older than age 60 years in the recipient group aged 60 to 69 years, which disappeared when using propensity score matching. Similar results were found in a study from Darie and colleagues^20^ where survival was inferior using donor lungs older than 65 year old in the univariate analysis but noninferior when using multivariate Cox regression. We conducted a Kaplan-Meier analysis for retransplantation-free survival in different recipient age groups (younger than age 60 years vs age 60 years or older) (Figure E1) and we did not see a significant difference between these groups when stratified for donor age (younger than age 70 years vs age 70 years or older). However, we believe our single-center cohort is not of sufficient size to perform further recipient analyses. We still believe that there are differences in the recipient population that we cannot acknowledge in a retrospective manner and therefore further studies are needed to account for the right allocation of available organs.

Furthermore, as clinicians, we must consider whether a statistically significant survival difference of approximately 10 percentage points at 5 years after LTx is clinically relevant considering the scarce options. We argue that these donors should be used preferentially to selected recipients where an inferior survival could be clinically accepted. However, in the rare, severely compromised recipient (eg, using a ventilator or receiving extracorporeal membrane oxygenation) we do not have the luxury of being too selective to achieve early survival in the sickest population.

### Study limitations

This is a retrospective study with its inherent limitations and potential bias, and it is possible that residual confounding remains despite having performed a propensity score-adjusted analysis. On the other hand, the utilization of Swedish personal numbers ensure that we do not have much missing data because we can follow patients nationally without loss of follow-up, although we do not know the cause of death.

## Conclusions

This study shows that 1-year retransplantation-free survival using grafts from donors older than age 70 years is comparable to those of younger donors, but long-term survival is inferior, which indicates that grafts from older donors should be used in selected patients undergoing LTx where the anticipated lower survival rate could be acceptable.

## Conflict of Interest Statement

G. Dellgren has received an institutional research grant from Astellas Europe A/S for the investigator-initiated ScanCLAD study (No. NCT02936505); an Abbott institutional research grant regarding a destination therapy study on left ventricular assist devices (No. NCT02592499); an institutional research grant from XVIVO for an industry-initiated study on organ preservation (NCT03991923); and is an XVIVO board member. All other authors reported no conflicts of interest.

The *Journal* policy requires editors and reviewers to disclose conflicts of interest and to decline handling or reviewing manuscripts for which they may have a conflict of interest.

## Figures and Tables

**Table E1:** Adjusted Cox regression to evaluate predictors associated with death or retransplantation

Variable	Value	n (%) events	Crude event rate (95% CI)	Adjusted model hazard ratio (95% CI)∗
Age per 10-y increase	≤median (57.7∗)	180 (52.0)	9.4 (8.1-10.9)	
	>median (57.7)	191 (56.5)	14.0 (12.1-16.1)	1.23 (1.13-1.34) *P* < .0001
Recipient sex	Male	195 (55.1)	12.8 (11.1-14.8)	1.17 (0.95-1.44) *P* = .14
	Female	180 (53.7)	10.2 (8.8-11.8)	
Blood type	O	134 (53.8)	10.7 (9.0-12.7)	
	A	186 (55.7)	11.8 (10.2-13.6)	1.10 (0.88-1.38) *P* = .40
	B	44 (57.9)	14.0 (10.2-18.8)	1.25 (0.88-1.77) *P* = .21
	AB	11 (40.7)	8.0 (4.0-14.2)	0.78 (0.42-1.45) *P* = .44
Recipient weight per 5-kg increase	≤median (68.0∗)	173 (49.4)	9.4 (8.1-10.9)	
	>median (68.0)	195 (59.1)	13.7 (11.9-15.8)	1.02 (0.99-1.06) *P* = .18
Recipient height per 5-cm increase	≤median (170.0∗)	186 (53.1)	10.4 (9.0-12.0)	
	>median (170.0)	185 (55.6)	12.4 (10.7-14.3)	0.98 (0.92-1.05) *P* = .53
BMI per 1-point increase	≤median (23.3∗)	170 (50.6)	9.4 (8.1-11.0)	
	>median (23.3)	198 (57.6)	13.7 (11.8-15.7)	1.02 (1.00-1.05) *P* = .039
Recipient smoking	No	132 (43.9)	9.3 (7.8-11.0)	
	Past use <6 mo	1 (50.0)	4.2 (0.1-23.2)	0.45 (0.06-3.22) *P* = .43
	Past use >6 mo	241 (62.8)	13.2 (11.6-15.0)	1.17 (0.93-1.48) *P* = .17
Recipient diagnosis	COPD	99 (64.3)	12.3 (10.0-15.0)	
	Alpha-1 antitrypsin deficiency	44 (66.7)	12.8 (9.3-17.2)	1.04 (0.73-1.48) *P* = .84
	Other	34 (42.5)	9.9 (6.8-13.8)	0.96 (0.63-1.46) *P* = .84
	Pulmonary vascular disease	22 (51.2)	10.4 (6.5-15.8)	1.11 (0.66-1.85) *P* = .69
	Cystic fibrosis	14 (27.5)	3.8 (2.1-6.4)	0.41 (0.22-0.78) *P* = .0065
	Pulmonary fibrosis	128 (53.3)	13.4 (11.1-15.9)	1.03 (0.79-1.36) *P* = .81
	Re-transplantation	14 (73.7)	14.7 (8.0-24.6)	1.26 (0.72-2.22) *P* = .42
	Other interstitial lung disease	20 (58.8)	12.9 (7.9-20.0)	1.01 (0.62-1.66) *P* = .95
LTx y (per 5-y increase)	≤median (2016∗)	272 (72.7)	11.1 (9.8-12.5)	
	>median (2016)	99 (31.9)	11.8 (9.6-14.4)	0.93 (0.82-1.05) *P* = .23
Respirator preoperative	No	357 (54.7)	11.5 (10.3-12.7)	
	Yes	16 (53.3)	10.5 (6.0-17.1)	0.90 (0.52-1.55) *P* = .71
ECMO preoperative	No	355 (54.3)	11.4 (10.3-12.7)	
	Yes	20 (58.8)	12.0 (7.3-18.6)	1.11 (0.68-1.83) *P* = .67
Dialysis preoperative	No	370 (54.5)	11.4 (10.3-12.7)	
	Yes	4 (80.0)	17.0 (4.6-43.6)	1.22 (0.39-3.83) *P* = .73
Urgency	Patient on extra-corporeal circulatory support	13 (72.2)	19.6 (10.4-33.5)	
	Urgent	4 (30.8)	5.8 (1.6-14.9)	0.43 (0.14-1.35) *P* = .15
	Not marked as urgent	358 (54.4)	11.4 (10.2-12.6)	0.58 (0.32-1.04) *P* = .07
Preoperative FEV1 (L)	≤median (1.0∗)	181 (56.6)	10.6 (9.1-12.3)	
	>median (1.0)	169 (53.1)	12.8 (10.9-14.8)	1.05 (0.89-1.23) *P* = .59
Percent of expected FEV1 (%)	≤median (33.2∗)	180 (56.4)	10.5 (9.0-12.1)	
	>median (33.2)	170 (53.3)	13.1 (11.2-15.2)	1.00 (1.00-1.01) *P* = .43
6-min walking test distance	≤median (276.0∗)	180 (58.8)	12.3 (10.5-14.2)	
	>median (276.0)	157 (51.3)	11.0 (9.3-12.8)	1.00 (1.00-1.00) *P* = .28
LTx procedure	Single LTx	100 (80.0)	14.7 (12.0-17.9)	
	Double LTx	275 (48.8)	10.6 (9.4-11.9)	0.88 (0.69-1.12) *P* = .30
Donor age (per 10-y increase)	≤median (5.2∗)	173 (50.0)	9.7 (8.3-11.3)	
	>median (5.2)	198 (58.6)	13.2 (11.4-15.2)	1.08 (1.01-1.15) *P* = .030
Donor age	<70 y	330 (53.7)	10.9 (9.8-12.1)	
	≥70 y	45 (60.0)	17.8 (13.0-23.8)	1.44 (1.04-1.98) *P* = .026
Donor sex	Male	179 (53.8)	11.6 (10.0-13.4)	
	Female	196 (55.1)	11.3 (9.8-13.0)	1.07 (0.86- 1.33) *P* = .54
Donor blood type	O	162 (53.3)	10.9 (9.3-12.8)	
	A	158 (55.2)	11.5 (9.8-13.5)	1.06 (0.85-1.32) *P* = .61
	B	42 (60.0)	13.8 (9.9-18.6)	1.23 (0.87-1.73) *P* = .25
	AB	3 (33.3)	5.6 (1.1-16.3)	0.51 (0.16-1.61) *P* = .25
Donor weight (per 5-kg increase)	≤median (73.5∗)	180 (52.8)	10.8 (9.3-12.5)	
	>median (73.5)	189 (55.6)	11.8 (10.2-13.6)	1.00 (0.97-1.03) *P* = .99
Donor height (per 5-cm increase)	≤median (172.0∗)	198 (54.4)	11.6 (10.0-13.3)	
	>median (172.0)	171 (53.9)	11.1 (9.5-12.9)	0.97 (0.92-1.02) *P* = .25
Donor BMI (per 1-point increase)	≤median (24.7∗)	191 (54.7)	11.5 (9.9-13.2)	
	>median (24.7)	177 (53.5)	11.1 (9.5-12.9)	1.01 (0.98-1.03) *P* = .58
Donor smoking	No	85 (39.4)	10.7 (8.5-13.2)	
	Yes	40 (42.6)	12.4 (8.8-16.8)	1.12 (0.77-1.64) *P* = .56
	Previous smoker	21 (38.9)	17.0 (10.5-26.0)	1.31 (0.80-2.14) *P* = .28
Donor cause of death	Spontaneous intracranial hemorrhage	231 (59.4)	12.2 (10.7-13.9)	
	Cerebral infraction	17 (53.1)	12.8 (7.5-20.6)	1.01 (0.62-1.65) *P* = .97
	Traumatic brain injury	22 (34.9)	10.0 (6.3-15.2)	0.75 (0.48-1.16) *P* = .20
	Cerebral anoxia after cardiorespiratory arrest	21 (27.6)	7.4 (4.6-11.3)	0.60 (0.38-0.94) *P* = .026
	CNS infection	6 (85.7)	21.2 (7.8-46.1)	1.70 (0.75-3.85) *P* = .20
	Other causes of death	78 (63.9)	10.7 (8.5-13.4)	0.92 (0.71-1.20) *P* = .55
Donor time in ICU (d)	≤median (2.1∗)	222 (66.5)	12.0 (10.5-13.7)	
	>median (2.1)	142 (41.9)	10.3 (8.7-12.1)	1.01 (0.97-1.04) *P* = .72
Waiting d, active	≤median (5.3∗)	192 (56.1)	11.7 (10.1-13.4)	
	>median (5.3)	178 (52.2)	10.9 (9.4-12.7)	1.00 (0.99-1.01) *P* = .65
Donation after circulatory death	No	373 (55.7)	11.5 (10.3-12.7)	
	Yes	2 (10.5)	7.2 (0.9-26.2)	0.51 (0.13-2.05) *P* = .34

*BMI*, Body mass index; *COPD*, chronic obstructive pulmonary disease; *LTx*, lung transplantation; *ECMO*, extracorporeal membrane oxygenation; *FEV1*, forced expiratory volume in 1 s; *CNS*, central nervous system; *ICU*, intensive care unit.

∗ Cox regression adjusted for age and sex.

**Table E2:** Propensity score-matched groups

	<70 y donor (n = 63)	≥70 y donor (n = 63)	*P* value	SMD
Recipient age (y)	60.2 ± 9.3 62.56 (26.70 to 74.31) n = 63	60.2 ± 10.2 63.38 (15.77 to 72.94) n = 63	.76	0.01
Recipient sex			.72	
Male	28 (44.4)	30 (47.6)		−0.06
Female	35 (55.6)	33 (52.4)		0.06
Recipient weight (kg)	66.9 ± 14.0 65.00 (33.50 to 104.00) n = 63	68.1 ± 14.0 67.00 (38.50 to 104.00) n = 63	.63	−0.09
Recipient height (cm)	166.8 ± 9.5 167.00 (145.00 to 186.00) n = 63	168.9 ± 8.2 168.00 (153.00 to 184.00) n = 63	.19	−0.24
Recipient BMI per 1-point increase	24.0 ± 4.3 23.50 (14.50-32.65) n = 63	23.9 ± 4.9 23.69 (15.87-32.72) n = 63	.93	0.02
Recipient blood type			.45	
O	23 (36.5)	29 (46.0)		−0.19
A	29 (46.0)	23 (36.5)		0.19
B	8 (12.7)	9 (14.3)		−0.05
AB	3 (4.8)	2 (3.2)		0.08
Recipient diagnosis			.55	
COPD	20 (31.7)	19 (30.2)		0.03
Alpha-1 antitrypsin deficiency	3 (4.8)	5 (7.9)		−0.13
Other	0 (0.0)	2 (3.2)		−0.26
Pulmonary vascular disease	3 (4.8)	4 (6.3)		−0.07
Pulmonary fibrosis	29 (46.0)	28 (44.4)		0.03
Retransplantation	2 (3.2)	2 (3.2)		0.00
Other interstitial lung disease	6 (9.5)	3 (4.8)		0.19
Recipient smoking			.56	
No	17 (27.0)	20 (31.7)		−0.10
Past use	46 (73.0)	43 (68.3)		0.10
Respirator preoperative			1.00	
No	62 (98.4)	62 (98.4)		0.00
Yes	1 (1.6)	1 (1.6)		0.00
ECMO preoperative			1.00	
No	60 (95.2)	60 (95.2)		0.00
Yes	3 (4.8)	3 (4.8)		0.00
Dialysis preoperative			.32	
No	62 (98.4)	63 (100.0)		−0.18
Yes	1 (1.6)	0 (0.0)		0.18
Last urgency			.48	
Patient on extra-corporeal circulatory support	3 (4.8)	1 (1.6)		0.18
Urgent	1 (1.6)	2 (3.2)		−0.10
Not marked as urgent	59 (93.7)	60 (95.2)		−0.07
Preoperative FEV1 (L)	1.3 ± 0.7 1.30 (0.30 to 3.22) n = 63	1.1 ± 0.6 0.97 (0.30 to 2.50) n = 58	.07	0.37
Percent of expected FEV1 (%)	44.4 ± 20.6 43.45 (10.58 to 97.00) n = 6 3	36.1 ± 16.9 34.31 (11.69 to 69.55) n = 58	.031	0.45
6-min walking test distance	279.0 ± 137.8 255.50 (20.00 to 604.00) n = 62	256.9 ± 110.4 244.00 (40.00 to 510.00) n = 55	.34	0.18
LTx y	2017 ± 4.7 2018 (2006 to 2024) n = 63	2017 ± 4.0 2018 (2009 to 2024) n = 63	.46	0.11
Donor sex			.56	
Male	18 (28.6)	21 (33.3)		−0.10
Female	45 (71.4)	42 (66.7)		0.10
Donor body weight (kg)	73.0 ± 14.4 70 (48 to 110) n = 63	73.9 ± 15.1 70 (51 to 140) n = 63	.74	−0.06
Donor height (cm)	167.7 ± 8.1 168 (153 to 187) n = 63	168.2 ± 8.5 168 (150 to 192) n = 63	.70	−0.07
Donor BMI per 1-unit increase	25.9 ± 4.4 25 (18 to 40) n = 63	26.0 ± 3.9 25 (20 to 41) n = 63	.93	−0.01
Donor blood type			.35	
O	27 (42.9)	35 (55.6)		−0.26
A	30 (47.6)	21 (33.3)		0.29
B	6 (9.5)	7 (11.1)		−0.05
Donor cause of death			.69	
Spontaneous intracranial hemorrhage	51 (81.0)	49 (77.8)		0.08
Cerebral infraction	3 (4.8)	4 (6.3)		−0.07
Traumatic brain injury	6 (9.5)	6 (9.5)		0.00
Other causes of death	3 (4.8)	4 (6.3)		−0.07
Donor smoking			.61	
No	31 (49.2)	28 (44.4)		0.10
Yes	3 (4.8)	2 (3.2)		0.08
Past use	7 (11.1)	9 (14.3)		−0.10
Missing	22 (34.9)	24 (38.1)		−0.07
Time in ICU donor (d)	3.0 ± 2.8 2 (−1 to 17) n = 62	2.7 ± 1.9 2 (1 to 11) n = 59	.58	0.13
Waiting d, active	118.9 ± 162.1 64 (2 to 986) n = 63	106.6 ± 110.1 66 (1 to 463) n = 63	.83	0.09
Donation after circulatory death			.65	
No	61 (96.8)	60 (95.2)		0.08
Yes	2 (3.2)	3 (4.8)		−0.08

Values are presented as mean ± SD, median (range), or n (%). *SMD*, Standardized mean difference; *BMI*, body mass index; *COPD*, chronic obstructive pulmonary disease; *ECMO*, extracorporeal membrane oxygenation; *FEV1*, forced expiratory volume in 1 s; *LTx*, lung transplantation; *ICU*, intensive care unit.
